# Pre‐Registration Nursing Student Experiences of International Mental Health Clinical Placement: A Scoping Review With Relevance to the Australian Context

**DOI:** 10.1111/inm.70221

**Published:** 2026-01-27

**Authors:** Alana Wilson, Renee Molloy, Adam Searby

**Affiliations:** ^1^ Monash University School of Nursing and Midwifery Melbourne Victoria Australia

**Keywords:** clinical placement, international nursing students, mental health nursing, nursing, psychiatric, student experiences, student placement, students, nursing

## Abstract

Clinical placement is an essential part of Australian pre‐registration nursing degrees and is mandated for all students to become registered as nurses. Clinical placement in mental health settings is important for preparing students to work with individuals with mental ill health and mental health conditions, with positive experiences during mental health placements reported to increase the desirability of mental health nursing as a career pathway. Given Australia's reliance on international students in the tertiary sector and nurses born in countries other than Australia, there is a dearth of research exploring the experiences of international pre‐registration nursing students on mental health clinical placement. This scoping review aimed to explore existing literature examining international student experiences of mental health clinical placements. Database searches of the CINAHL, Emcare, MEDLINE, Scopus and PsycINFO databases found no literature specifically examining the experience of international nursing students in Australia on mental health clinical placements. The search was expanded to conceptually analyse published papers (*n* = 25) exploring nursing student experiences of mental health nursing placement from around the world. Themes that emerged were fear and apprehension, skills, knowledge and attitude changes, managing own emotions and uncomfortable experiences, translating theory to practice, and opportunities for active learning and support needs. Our review highlights an urgent need for research into the experiences of Australian pre‐registration international nursing student experiences of mental health clinical placements, both to understand the challenges this student cohort experiences, and to improve the recruitment of international nursing students to the mental health nursing specialty.

## 1 Introduction

The global nursing shortage is a critical issue affecting healthcare systems around the world. Factors such as, an aging population, a rise in chronic health conditions, and the recent pandemic have resulted in the demand for nursing care to surge beyond the current supply of qualified professionals (Hurley et al. 2022). This shortage places significant strain on healthcare systems and directly impacts both the quality and accessibility of patient care (Buus et al. 2020) and poses challenges to addressing the current mental health crisis. Currently, only 6% of the Australian nursing workforce identifies as working in mental health (Australian Institute of Health and Welfare 2024), and with this comes a predicted shortage of mental health nurses (Buus et al. 2020; Hurley et al. 2022). Mental health nurses provide speciality care including assessment, treatment, and support to promote recovery and well‐being; working closely with patients to manage symptoms, develop coping strategies, and connect them with resources, fostering a holistic approach to mental health care (Felton et al. 2018; Hurley et al. 2022). As nurses account for over half of the total health industry employment in Australia (Australian Institute of Health and Welfare [AIHW] 2024), they are well positioned to recognise and respond to the mental health needs of all people regardless of the primary reason for treatment.

One strategy to address the critical nursing workforce shortage in Australia has been through the migration of skilled nursing staff from other countries and increasing international student presence in local pre‐registration nursing courses (Australian Government Department of Health and Aged Care 2024). The Australian Government Department of Education (2025) defines an international student as one who is in Australia on a temporary visa and studying at a higher education institution or provider. Currently, one in five nurses in Australia entered the profession as international students, and approximately 41.8% of initial registrations to the nursing profession are either overseas‐trained applicants or international students (Australian Institute of Health and Welfare 2024; Australian Health Practitioner Regulation Agency [AHPRA] 2024), showing a reliance on international students to bolster workforce numbers.

## 2 Background

Clinical placement, a type of work‐integrated learning where students spend time in a clinical environment to consolidate their theoretical knowledge, plays a pivotal role in preparing students for their future working conditions by bridging the gap between theoretical knowledge and real‐world application knowledge (Anyango et al. 2024). These hands‐on experiences allow students to immerse themselves in the complexities of clinical environments, develop essential skills, and adapt to healthcare settings (Patterson et al. 2016). International students are reported to find clinical placements challenging due to cultural and language barriers that impact their engagement and confidence in clinical settings, with cultural differences reported to create misunderstandings around workplace norms and patient care expectations, while language barriers complicate communication with patients and staff (Hansen et al. 2021; Mitchell et al. 2017; McKitterick et al. 2021). Further, limited social support can add to feelings of isolation, making it harder to navigate and adapt to the demands of placement (Jeong et al. 2011; Vardaman and Mastel‐Smith 2016). These differences create additional stressors for international students and places students at risk of abandoning their nursing education and can carry over into confidence issues upon graduation and transition to the health workforce (Khawaja Chan and Stein 2017; McKitterick et al. 2021).

In Australia, pre‐registration nurses are educated in comprehensive programs, and although accreditation standards for nursing and midwifery programs do not stipulate requirements for students to complete a mental health clinical placement, most programs do include one (Australian Nursing and Midwifery Accreditation Council 2019). Clinical placement in mental health settings is important for preparing nurses to work with individuals with mental illness as it improves knowledge and skills, including developing rapport and therapeutic relationships, reduces stigma and increases empathy (Foster et al. 2021). Furthermore, positive experiences on mental health placement can increase the desirability of mental health nursing as a career pathway, a crucial factor for recruitment as mental health nursing is not a popular career pathway for newly qualified nurses (Happell et al. 2015; Stuhlmiller and Tolchard 2019). Despite these benefits, there are unique challenges associated with mental health placements. For example, nursing students in Australia (Happell et al. 2015) and abroad (Alhamidi and Alyousef 2023; Masutha et al. 2023) experienced fears of violence and personal safety going into mental health placement. International students may face additional challenges on mental health placement as cultural beliefs have been found to influence nursing students' attitudes towards mental health conditions, including understanding and treatment interventions and stigma (Hansen et al. 2021).

Previous literature reviews have explored clinical placements in mental health settings (Happell et al. 2015), and the impact of pre‐registration student culture on their learning about mental health (Hansen et al. 2021). However, no reviews have explored the experiences of Australian international students on mental health placement. Therefore, the aim of this review is to map what is known about the experiences of pre‐registration student nurses on mental health clinical placements in an international context. In this paper, we define pre‐registration students as those completing an undergraduate or master's level qualification that enables them to obtain their initial registration as a nurse.

## 3 Methods

This scoping review followed the five steps recommended by Arksey and O'Malley (2005) (1) identifying the review aims, (2) identifying relevant studies, (3) study selection, (4) charting the data, and (5) collating, summarising and reporting the results. Review findings are reported in accordance with the Preferred Reporting Items for Systematic Reviews and Meta‐Analyses Extension for Scoping Reviews (PRISMA‐ScR) (Tricco et al. 2018).

### 3.1 Identifying the Review Aims

The original aim of this review was to map literature on the mental health placement experiences of international nursing students in Australian universities. However, an exploratory search of CINAHL (EBSCOhost) yielded no published literature that specifically explored this phenomenon. In addition, a search of the Cochrane Database of Systematic Reviews, JBI Evidence Synthesis and Open Science Framework was conducted, and no published or in‐progress systematic or scoping reviews on the topic were identified. To explore the ‘conceptual boundaries’ of the topic (Peters et al. 2015), the search was broadened to include all countries. By encompassing the overall undergraduate student nurse experience of clinical placements in a mental health clinical setting, we were able to explore the applicability and future research directions of these findings to the international student cohort. There were four questions guiding the design of the literature search and conduct of this scoping review:

1. What preconceived ideas do nursing students have about mental health clinical placement and where do these preconceived ideas come from?
2. In what ways does mental health placement transform pre‐registration nursing students?
3. What were the issues that emerged during clinical placement in a mental health clinical setting that caused concern for students?
4. What factors would contribute to a more effective mental health clinical placement?

### 3.2 Identifying Relevant Studies

CINAHL (EBSCOhost), Emcare (Ovid), MEDLINE (Ovid) and PsycINFO were searched between July and August 2024. Key terms were identified using the PICo framework (Schardt et al. 2007), and inclusion criteria were selected in keeping with this framework (see Table 1 for inclusion and exclusion criteria):

- Population—Pre‐registration nursing students
- Interest—Clinical placement
- Context—Mental health settings, not limited to country

**TABLE 1:** Inclusion and exclusion criteria.

Inclusion criteria	Exclusion criteria
Pre‐registration nursing student	Postgraduate/post‐registration nursing student
Clinical placement	Non‐nursing focus
Mental health setting/traditional	Simulation/immersion
Any country	Curriculum design
Date range 2000 and beyond	Publication not in English language
Primary research	Literature review, protocols, and other non‐primary research, such as discussion papers and letters to the editor

Using the key terms, an initial search strategy for CINAHL was established in consultation with a university librarian (See Supporting Information). This strategy was then adapted for the remaining three databases. All countries were included, with papers limited to those published in English. Peer‐reviewed papers were included from 2000 to 2024. The rationale for this date range was considered as the landscape and language of mental health nursing and care has changed within the last 25 years. Changes in legislation, for example new Mental Health Acts, and the move towards consumer‐led care have altered the contexts of clinical placement.

### 3.3 Study Selection

**FIGURE 1:**
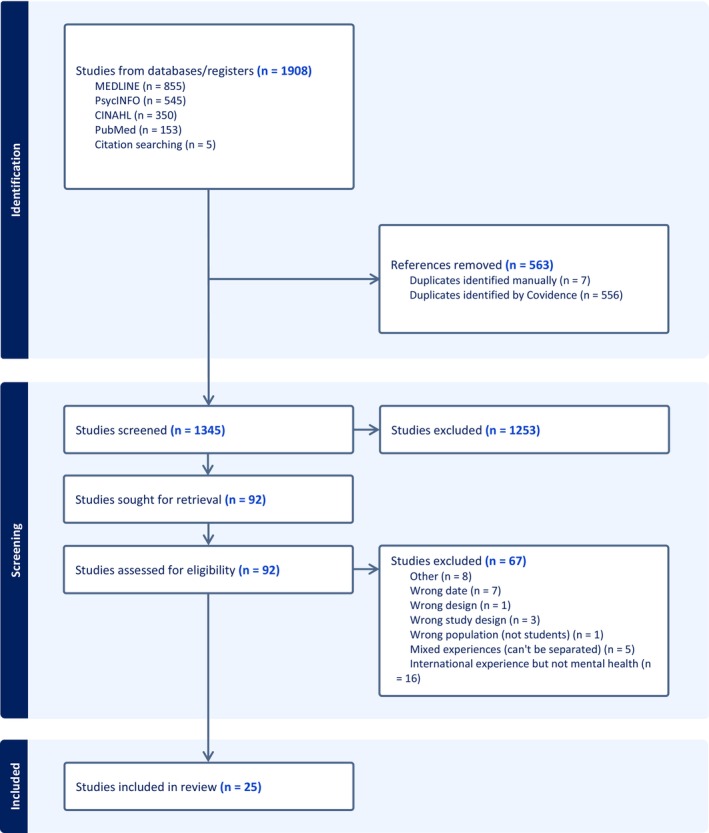
PRISMA study flow diagram.

Following database searching, all identified articles (*n* = 1908) were uploaded to Covidence (Veritas Health Innovation, Melbourne, Australia) for screening. After removal of duplicate papers, 563 results remained for title and abstract screening against the inclusion and exclusion criteria. Following independent title and abstract screening by Author One and Author Two, 92 sources were included for full text screening. These sources underwent full text screening against the inclusion and exclusion criteria by Author One and Author Two independently. Conflicts were resolved through discussion with Author Three. Following this step, 25 papers remained for data extraction (see Figure 1).

### 3.4 Charting the Data

The data extraction function on Covidence was used to extract the following data: article author and date, country of origin, study design, the study sample size, the aim of the study, main findings of the study and any limitations and strengths. This data extraction stage was completed in full by Author One with Author Two and Author Three each extracting 10% each to ensure validity (Levac et al. 2010). A summary of the extracted data for the studies included in this review is shown in Table 2.

**TABLE 2:** Characteristics of selected studies.

Author, date, country	Study design and sample	Aim of study	Findings
Acı et al. (2022), Turkey	Qualitative: phenomenological method, focus group interviews (*n* = 25 undergraduate nursing students)	To examine the clinical practice experiences of psychiatric and mental health nursing students	The use of images and warnings may create prejudice and should be avoided prior to clinical placement Satisfaction and positive attitude changes were noted post‐placement The authors recommend increasing ‘in‐clinic’ time to improve satisfaction and attitudes towards those with mental illness, and to provide mental health courses in the first year of study (pre‐placement)
Alhamidi and Alyousef (2023), Saudi Arabia	Qualitative: semi structured focus groups (*n* = 20 female undergraduate nursing students)	To identify factors that mediate positive and negative perceptions of undergraduate nursing students towards nursing placements in mental health care	Participants acknowledged the educational significance of their mental health clinical experience for consolidating and improving their theoretical knowledge and developing new skills All participants in both focus groups noted the harm of preconceived ideas and stigma, and the negative effect this thinking may have on patients Participants reported that apprehension and fear was experienced at the beginning of the course placement. Fear of physical danger to themselves or patients, upset them Participants reported becoming highly anxious about individuals with delusions and hallucinations or other mental health problems. However, after interacting with individuals suffering from hallucinations in the clinical area, participants in the focus groups found that their empathy levels improved
AL‐Sagarat et al. (2015), Jordan	Quantitative: non‐experimental, descriptive survey design (*n* = 72 undergraduate nursing students)	To examine the impact of clinical placement on undergraduate nursing students and their confidence	Clinical placements significantly improved the perceived confidence of participants in mental health settings, including conducting examinations and creating care plans There was a statistically significant increase in overall confidence scores post‐placement Participants showed increased confidence in their knowledge of medications used in mental health settings after the clinical placement
Al‐Zayyat and Al‐Gamal (2014), Jordan	Quantitative: Descriptive, longitudinal survey design conducted at two time points (*n* = 65 undergraduate nursing students)	To identify the degrees of stress, types of stressors and coping strategies used by undergraduate nursing students during their mental health placements	The highest reported stressors were taking care of patients, stress related to teachers and nursing staff, and from university workload, including assignments The authors postulate that stress in taking care of patients may be due to negative attitudes towards individuals with mental illness, in addition to participants encountering patients with complex biopsychosocial needs The most utilised strategy to manage stress was problem solving
Ashipala et al. (2023), Namibia	Qualitative: descriptive exploratory design, semi‐structured interviews (*n* = 15 undergraduate nursing students)	To explore and describe the experiences of nursing students during their mental health clinical training at a general hospital in Namibia	Participants reported positive learning experiences during their mental health clinical placements, gaining practical skills and confidence The absence of appropriate mental health units and a lack of trained psychiatric nurses to supervise them were significant challenges faced by students The authors recommend students are provided with placement opportunities in mental health units to improve their learning and skills and attitudes
Demir and Ercan (2018), Turkey	Qualitative: phenomenological approach, face to face interviews (*n* = 15 undergraduate nursing students)	To evaluate nursing student's clinical experiences with mental health patients during their first clinical placement	Themes emerged around stigma, and at times over‐identification of MH symptoms in themselves and family/friends, and previously held beliefs held back initial learning After completing their theoretical courses, participants did not feel safe entering mental health clinical placements, with the authors postulating that theoretical learning was insufficient to break stigma Fear was reduced and self‐confidence increased through interactions with individuals in the clinical environment
Foster et al. (2021), Australia	Qualitative, interpretive inquiry using focus groups (*n* = 31 undergraduate nursing students)	To explore nursing students' experiences of traditional mental health clinical placements and how those experiences influenced their practice and understandings of recovery from mental illness	Through positive placements, participants came to see patients as people rather than just diagnoses Peer‐support workers were important in helping students develop a better understanding of recovery and mental illness The authors conclude that nursing students need to be prepared and supported to deal with the potential for vicarious trauma during mental health clinical placements
Gonella et al. (2020), Italy	Quantitative: secondary analysis of a national, cross‐sectional study performed between 2015 and 2016 (*n* = 9607 undergraduate nursing students)	To explore nursing students' perceptions of their clinical learning experiences in the mental health setting	Mental health settings provided more learning opportunities, positively influencing students' perceived competence and interest in working in these settings The findings reveal concern that exposing students to mental health clinical environment early in their degree could increase distress and contribute to the development of negative attitudes towards mental illness Participants felt supported during placement, especially during difficult situations; the authors postulate that overall, these positive learning environments lead to students being attracted to mental health nursing
Granskär et al. (2001), Sweden	Qualitative: grounded theory analysis, individual interviews (*n* = 11 undergraduate nursing students)	To generate a theoretical model of nursing students' experience in their first professional encounter with people having mental disorders	Feelings of uncertainty and negative emotion concerning individuals with mental illness were common among participants before mental health training Where participants could not form therapeutic relationships with patients, they felt helpless and rejected
Günaydin and Arguvanli Çoban (2021), Turkey	Qualitative: phenomenological design using focus group interviews (*n* = 40 4th year undergraduate nursing students)	To identify experiences of nursing students on placement in mental health clinics	Participants described difficulty in translating their theoretical knowledge into practice Participants also described a lack of role model nurses and teaching staff within the clinical environment The authors conclude that there is a need for supportive interventions for students to cope with negative emotions reported by all students in the early days of their mental health placement. These emotions included fear, concern, anxiety, excitement, alienation and loneliness
Happell (2008), Australia	Mixed: Observational study, survey design, cross‐sectional, multi‐site (*n* = 703 undergraduate students)	To determine variables that impact student satisfaction with clinical placement	Participants reported high levels of satisfaction, particularly if they were welcomed, orientated efficiently and were supported Placement satisfaction was more positively influenced if placement was longer and participants had more time and supervision and support from preceptors Community placements were more highly regarded by participants than those conducted in inpatient units
Henderson et al. (2007), Australia	Quantitative: cross‐sectional survey (*n* = 146 undergraduate nursing students)	To examine the factors that contribute to satisfaction of a clinical placement in mental health and at what level	Support from clinical staff and active involvement in patient care were described as key factors for a positive clinical placement experience Participants reported higher satisfaction with inpatient mental health settings compared to community settings Participants described inpatient placements as providing more opportunities for engagement and preparation for practice as beginning mental health practitioners
Hung et al. (2009), Taiwan	Qualitative: phenomenological approach using individual interviews (*n* = 12 undergraduate nursing students)	To understand the experiences of students' mental health clinical placement and use those findings to improve teaching strategies	The results indicate that clinical practice helps reduce stigma and fear among psychiatric nursing students, leading to more positive attitudes towards patients The authors conclude a combination of theoretical and practical training is essential for developing professional and self‐confident psychiatric nursing students, and that understanding student perceptions during initial clinical experiences is crucial for improving educational programs
Hwang et al. (2018), Korea	Qualitative: open ended survey and qualitative content analysis of journal entries made during mental health placement (*n* = 59 undergraduate nursing students)	To explore types of events or issues that senior nursing students chose to reflect upon in their critical reflective journals during their five‐week psychiatric mental health nursing clinical placement	Journal entries by participants reflected on boundaries in therapeutic relationships, responses to patient symptoms, and their own attitudes and biases towards mental illness Journaling indicated participants perceived an increase in competency in mental health nursing Critical reflective journaling enhanced participant self‐reflection, motivation, and understanding, with the authors postulating that reflective journalling led to improved competency in mental health nursing
Janse Van Rensburg et al. (2012), South Africa	Qualitative: exploratory descriptive design using focus group interviews (*n* = 13 undergraduate nursing students), and “naïve sketches” (*n* = 11)	To explore and describe the experiences of student nurses and gain a deeper understanding of these experiences to create awareness of the support needed	Participants experienced intense emotional discomfort initially when working with individuals with mental ill health, described by some as emotionally and physically draining Participants moved through ‘stages’ when engaging with individuals with mental ill health: experiencing empathy, generating a sense of gratitude and hope, acknowledging the potential of the individual with mental ill health and the discovery of resilience within themselves
Karimollahi (2012), Iran	Qualitative: phenomenological design using individual interviews (*n* = 13 undergraduate nursing students)	To investigate the clinical experiences of nursing students undertaking placement on a mental health unit	Participants initially experienced anxiety due to preconceived notions about mental health and patients, including fear of violence, fear of the unknown and anxiety. These preconceived notions were gained from the media, peers, and society Over time, participants developed positive attitudes and satisfaction with their clinical experience as they adapted and engaged with patients Participants were reported to be overly dependent on their instructor in the environment, “shirking” their own duty and blaming the instructor for not teaching them
Kidd and Tusaie (2004), USA	Qualitative: thematic analysis of poems written by students about their mental health placement (*n* = 52 student poems)	To present an interpretive study in which poetry writing was used to know the lived experience of nursing students during their first mental health clinical	Five themes emerged from participant poems: fears about personal safety and competence, empathy for clients, normalisation of mental illness, the student as a wounded healer, and personal metamorphosis Poetry writing helped participants challenge and alter their initial beliefs about mental illness
Koskinen et al. (2011), Finland	Qualitative: analysis of written narratives of experiences on mental health nursing placement (*n* = 20 student summaries)	To describe the components of nursing student's clinical placement experiences in mental health and using those perspectives to improve mentoring and learning for future students	Participants initially had negative attitudes towards mental health settings, which improved through exposure to challenging care situations Participants developed increased self‐awareness and self‐esteem, improved nurse–patient relationships, and learned new mental health care methods
Lim et al. (2020), Singapore	Quantitative: cross‐sectional survey (*n* = 144 undergraduate nursing students)	To examine undergraduate nursing students' perceptions towards mental health clinical placement experiences and assess the degree of clinical confidence among nursing students	Overall, participants showed a positive attitude towards mental health clinical placements, however initially experienced anxiety towards mental illness and negative stereotypes Where stereotypes were harboured by participants, there was less intention to pursue mental health nursing as a career Participants were least confident in handling verbal and physical aggression
Mansouri and Darvishpour (2024), Iran	Qualitative, descriptive design using semi‐structured interviews (*n* = 18 undergraduate nursing students)	To identify metaphors used by undergraduates on their first mental health clinical placement	Five categories emerged from the participants' metaphors from their first encounters of individuals with mental illness: experience is similar to fear mixed with excitement, the patient is similar to an errant human, a psychiatric hospital is similar to a prison, the nurse is similar to a prison guard and the clinical instructor is similar to a supporter, sympathetic and knowledgeable friend The sense of fear decreased as the placement progressed, with views reported as changing from fear to wonder and excitement. However early emotions experienced also included worry, anxiety, alienation and loneliness
Moagi et al. (2013), South Africa	Qualitative: focus group interviews (*n* = 29 undergraduate nursing students)	To explore nursing students' experiences of mental health placement and how to promote their own mental health in the workplace	Participants described feelings of fear, anxiety and apprehension before their placement, describing mental health nursing as a new experience that created emotional discomfort for them Participants found it difficult to observe verbal and physical aggression exhibited by some patients However, some described mental health placement as a “life changing” process, resulting in growth on multiple levels
Shen et al. (2023), China	Qualitative: semi‐structured interviews (*n* = 22 undergraduate nursing students)	To explore the experiences of undergraduate nursing students on their MH clinical placement and elicit their potential future career prospects in the specialty	Participants had preconceived bias and stereotypes towards mental health prior to placement; these were reported to be due to media and negative public perceptions Fear decreased through the placement experience, participants also felt that they were able to communicate better with people with mental illness as the placement progressed Renewed understanding of mental health but students felt powerless to help Most students expressed that they had no interest in working in mental health after graduation
Slemon et al. (2018), Canada	Qualitative: narrative enquiry using semi‐structured individual interviews (*n* = 15 undergraduate nursing students)	To describe how nursing students reflect on their experiences on clinical placement, understand the social constructs that influence those experiences and how students reflect on future care of people with mental health conditions	Fear of the unknown and apprehension was experienced across the cohort Negative views about people with mental health issues are held by students until they undertake clinical placement in a mental health setting On placement, participants witnessed issues around power and disempowerment, and practices that they felt were unsafe and morally distressing
Song (2015), South Korea	Qualitative: thematic analysis of semi‐structured interviews (*n* = 11 undergraduate nursing students)	To examine nursing student perspectives of MH clinical placement since the change in the country's Mental Health Act	Participants started with very negative views about mental health before starting placement. These views came from personal experience, education towards mental health placement which was perceived as negative, and fearful scenes in the media Participants described the work undertaken by mental health nurses as ‘negative:’ this occurred due to observing activities such as patient restraint or the administration of medication
Zhang et al. (2021), China	Qualitative: phenomenological approach using semi‐structured interviews (*n* = 13 undergraduate nursing students)	To explore the experience of undergraduate nursing students during their clinical placement to determine if it impacted on a future career in the specialisation	Participants described negative stereotypes about mental illness, such as individuals with mental illness always being prone to violence and dangerous behaviour. This often manifested as fear that they would be harmed during the mental health placement Participants gained confidence through the placement; this included developing empathy for those with mental illness and an improved attitude towards Despite positive experiences, psychiatric nursing is not considered an ideal career choice by nursing students

### 3.5 Collating, Summarising and Reporting the Results

Extracted data was exported from Covidence (Veritas Health Innovation, Melbourne, Australia) into Microsoft Word and displayed in table format to enable a visual summary of the study characteristics. A deductive approach, directed by Saldana's Structural Coding method (Saldaña 2013) was used to analyse included study findings and code them to the four questions guiding this scoping review: (1) What preconceived ideas do nursing students have about mental health clinical placement and where do these preconceived ideas come from? (2) In what ways do MH placement transform pre‐registration nursing students? (3) What were the issues that emerged during clinical placement in a mental health clinical setting that caused concern for students? and (4) What factors would contribute to a more effective mental health clinical placement?

## 4 Results

Of the 25 papers extracted for this review, 19 used a qualitative methodology, five used a quantitative methodology and one used a mixed method approach in the studies. Most papers located in the search were published within the last 10 years. There were nine Australian papers, followed by three from Turkey, two from Jordan, two from South Korea and one each from Canada, Sweden, Finland, Saudi Arabia, Norway, Taiwan, Singapore, Italy, Namibia and the United States. A summary of the main themes resulting from the structural analysis of the included papers is shown in Table 3.

**TABLE 3:** Summary of review results.

Research question	Theme
What preconceived ideas do nursing students have about mental health clinical placement and where do they come from?	Fear and apprehension exacerbated by preconceived ideas presented to from mass media and lecturers Individuals with mental illness are violent
2 In what ways do MH placement transform pre‐registration nursing students?	Skills: Communication Knowledge: Deeper understanding Attitudes: Empathy
3 What were issues that emerged during clinical placement that caused concern for students?	Managing own emotions and uncomfortable experiences Translating theory to practice
4 What factors would contribute to a more effective mental health clinical placement?	Importance of settings Opportunities for active learning Support

### 4.1 What Preconceived Ideas Do Pre‐Registration Nursing Students Have About Mental Health Clinical Placement, and Where Do They Come From?

#### 4.1.1 Fear and Apprehension Presented to Students From Mass Media and Lecturers

Participants described fear and apprehension prior to their mental health clinical placement and attributed this to a number of sources, including the media, society and educational settings (Alhamidi and Alyousef 2023; Karimollahi 2012; Shen et al. 2023; Song 2015). Several participants felt that they commenced mental health placement with negative views and biases about mental health disorders and mental health consumers (Hung et al. 2009; Hwang et al. 2018; Karimollahi 2012; Shen et al. 2023; Song 2015) because of how the media sensationalises topics such as electroconvulsive therapy and the use of physical restraint of people with mental illness (Alhamidi and Alyousef 2023; Demir and Ercan 2018; Song 2015). Two studies reported that this sentiment is amplified if the clinical setting is held in outdated or older buildings, echoing the dramatic background used on film sets; thus, precipitating feelings and preconceived ideas for students who were inexperienced and unversed in mental health (Hung et al. 2009; Shen et al. 2023).

Theoretical preparation in pre‐registration nursing programs was also described in some studies as contributing to mental health stigma (Demir and Ercan 2018; Slemon et al. 2018). Song (2015), and Acı et al. (2022), note that language used by lecturers in higher education contributed to this, especially when teaching about the early history of mental health without context and without showing modern alternatives to treatment. Tales of patients attacking nurses, being taught to be alert for potential harm and the need for self‐defence were embedded into these narratives, instead of discussing and eliminating stigmatising misconceptions before students enter the clinical placement environment (Acı et al. 2022).

#### 4.1.2 People With Mental Health Disorders Are Violent

The belief that individuals with mental illness would be dangerous and potentially attack them was shared among participants in several studies (Alhamidi and Alyousef 2023; Demir and Ercan 2018; Karimollahi 2012; Kidd and Tusaie 2004; Lim et al. 2020; Mansouri and Darvishpour 2024; Moagi et al. 2013; Zhang et al. 2021). Demir and Ercan (2018) likewise reported similar accounts from students who, at the commencement of their placement, held the belief that individuals with mental illness were dangerous, hostile and prone to unpredictable violence. Students expressed fear both of physical danger from the consumers and a fear that consumers would hurt themselves or that they would witness harm to other consumers (Kidd and Tusaie 2004). Participants reported concerns about their personal competence and their ability to effectively communicate in situations where verbal or physical aggression occurred (Demir and Ercan 2018), as well as doubts of ability to cope with the level of exposure to assumed danger before commencing their placement (Kidd and Tusaie 2004; Mansouri and Darvishpour 2024). Alhamidi and Alyousef (2023) observed how participants feared consumers becoming increasingly violent if they were unable to communicate with or care for them adequately or made an error in their care. This led to students holding back, being reserved, and keeping their distance from consumers early in the placement. Students were reported by Slemon et al. (2018) to perpetuate narratives of violence in the retelling of experiences from peers who had previously been on placement. However, incoming students to the placement were able to resist those narratives and formulate their own perspectives during their own time on placement.

### 4.2 In What Ways Do Mental Health Placement Transform Pre‐Registration Nursing Students?

#### 4.2.1 Skills: Communication

Mental health clinical placements transform pre‐registration nursing students by deepening their understanding of mental health care and fostering critical skills in communication (Koskinen et al. 2011). Communication skills were enhanced within the pre‐registration student cohorts (Alhamidi and Alyousef 2023; AL‐Sagarat et al. 2015; Demir and Ercan 2018; Granskär et al. 2001; Koskinen et al. 2011; Shen et al. 2023; Slemon et al. 2018) when there was a deeper understanding of how to support a person with mental health concerns and provide more recovery‐oriented care (Patterson et al. 2016; Perlman et al. 2017). This was achieved as students gained confidence in managing complex emotional and behavioural situations or were involved in discussions of what they were observing (Lim et al. 2020). Additionally, Janse Van Rensburg et al. (2012), noted that students experienced personal transformation through deeper emotional engagement and connection with consumers. These reported insights, therefore, contributed to improved skills in communication with individuals experiencing hallucinations and delusions; students learnt how to better communicate with empathy and how to navigate conversations around delusions without reinforcing them (Alhamidi and Alyousef 2023).

#### 4.2.2 Knowledge: Deeper Understanding

Clinical practice was reported by studies included in this review as crucial for improving nursing students' practical skills (Acı et al. 2022; Alhamidi and Alyousef 2023; AL‐Sagarat et al. 2015; Ashipala et al. 2023). Slemon et al. (2018) notes that any negative views about mental health and consumers are not likely to shift until students undertake clinical placement. Demir and Ercan (2018) suggest that with increased knowledge and exposure to placement, students could reduce their fears when they realised they could develop therapeutic relationships and communication skills. Furthermore, Koskinen et al. (2011) noted that exposure to consumers and care provision in the clinical setting provided the gateway to changing previously held beliefs and was instrumental in increasing confidence, competence and practical skills (Moagi et al. 2013).

As noted by Lim et al. (2020), a willingness to interact with individuals with mental illness is a strong predictor of students' intentions to work in mental health settings. For some students, witnessing moments of a consumer's personality and individuality, especially if it had been a consumer they had feared the most, was instrumental in students developing rapport and relationships (Song 2015). Students were reported to experience significant personal growth during their clinical placements, with many reporting a positive shift in their attitudes towards mental health nursing. Exposure to real‐world settings helps to demystify mental health care, allowing students to see firsthand the complexity and value of this area of nursing (Alhamidi and Alyousef 2023; Ashipala et al. 2023; Gonella et al. 2020; Karimollahi 2012; Lim et al. 2020; Mansouri and Darvishpour 2024; Moagi et al. 2013; Perlman et al. 2017; Zhang et al. 2021).

#### 4.2.3 Attitudes: Empathy

Through empathetic engagement, students become more attuned to the challenges faced by individuals with mental health conditions, moving beyond stereotypes and misconceptions (Janse Van Rensburg et al. 2012; Alhamidi and Alyousef 2023; Hung et al. 2009; Karimollahi 2012). They were also described as confronting and reducing personal biases about mental health, leading to a more compassionate approach and developing skills to validate consumers' feelings (Kidd and Tusaie 2004; Patterson et al. 2016). This shift encourages the rejection of discrimination and stigma, as students come to see patients as individuals deserving of respect and dignity (Alhamidi and Alyousef 2023; Hung et al. 2009; Janse Van Rensburg et al. 2012; Kidd and Tusaie 2004; Mansouri and Darvishpour 2024). By interacting with patients directly, using active listening, students gain insight into the impact of social stigma on mental health (Zhang et al. 2021), reinforcing the importance of providing non‐judgmental, inclusive care and creating safe spaces for consumers (Demir and Ercan 2018). This evolution in understanding prepares students to advocate for patients' rights and contribute to a more empathetic, stigma‐free healthcare environment with students feeling resolved at the end of their placement to counter misinformation and combat stigmatisation (Alhamidi and Alyousef 2023). These experiences prepare students to work holistically, emphasising the significance of patient‐centred care and therapeutic relationships.

### 4.3 What Were Issues That Emerged During Clinical Placement That Caused Concern for Students?

#### 4.3.1 Managing Own Emotions and Uncomfortable Experiences

During clinical placements students often encounter a range of emotional and educational challenges, particularly when dealing with therapeutic communication in mental health settings (Granskär et al. 2001; Janse Van Rensburg et al. 2012). Many students experienced heightened anxiety when interacting with patients exhibiting symptoms like delusions and hallucinations, which left them feeling unprepared and overwhelmed. This anxiety often led to psychological fatigue, as students invested significant emotional energy in managing these interactions (Demir and Ercan 2018). Similarly, Granskär et al. (2001) and Janse Van Rensburg et al. (2012) found that students often faced emotional discomfort and uncertainty when navigating these situations, which can exacerbate their feelings of helplessness, fear, and frustration.

For many students, witnessing practices that they felt were unsafe or ethically concerning; particularly when issues of power and disempowerment arise in patient care, was a morally distressing experience, particularly for students who felt powerless to intervene (Slemon et al. 2018). Song (2015) described how witnessing the use of physical restraint to administer medications by force profoundly affected nursing students, creating a resolve that mental health nursing would not be career path for them. In some cases, students' negative experiences reinforced confirmation biases, shaping their beliefs and attitudes towards mental health care (Granskär et al. 2001).

Another aspect of concern for students was their tendency to over‐identify with the mental health symptoms they observe. When students recognised similar issues in themselves or their family members this further added to their emotional burden (Demir and Ercan 2018; Song 2015) and deepened avoidant feelings around future mental health career paths.

#### 4.3.2 Translating Theory to Practice

Translating theoretical knowledge into practical skills was described as being of significant difficulty among students (Günaydin and Arguvanli Çoban 2021; Janse Van Rensburg et al. 2012). Günaydin and Arguvanli Çoban (2021). The theory to practice gap was noted by (Mansouri and Darvishpour 2024), when students were surveyed in the post clinical placement period and continued to view nurses as prison guards as well as holding beliefs that consumers are sinners; though there was, by contrast, an increase in reported compassion towards consumers. Song (2015) and Koskinen et al. (2011), examined reasons why these ongoing held beliefs occurred post placement and consider that it could be attributed to social‐cultural context. Another aspect to contribute to the issues around translating theory to practice is the absence of appropriate mental health units and trained mental health professionals, as noted by Ashipala et al. (2023).

### 4.4 What Factors Would Contribute to a More Effective Mental Health Placement?

#### 4.4.1 Importance of Settings

Henderson et al. (2007) found that inpatient settings were rated more favourably than community settings by nursing students, as inpatient placements provided more opportunities for direct engagement and hands‐on skills preparation. Happell (2008) reported contrasting findings: community settings received higher satisfaction ratings from students. This comparison highlights the complexity of students' placement experiences and the need for further investigation into what factors contribute to satisfaction and learning in different clinical settings. The importance of settings was later brought into focus in (Song 2015), when students commented that the old hospital building where their placement was held instigated feelings of fear with reports of higher levels of anxiety and increased negative bias towards mental health than those who practised their clinical placement in newer facilities was also recognised by this study.

#### 4.4.2 Support

Foster et al. (2021) indicated that nursing students need to be more prepared and supported to deal with the potential for vicarious trauma during clinical placements and to manage negative emotions, including role‐modelling to improve outcomes (Günaydin and Arguvanli Çoban 2021). Suggested strategies to support students' well‐being include use of reflective practices, including the use of journaling to assist in facilitating student learning, improved competency and motivation in mental health nursing skills (Hwang et al. 2018). Kidd and Tusaie (2004) took a different approach and found that the use of more artistic measurement in the form of poetry writing as a form of reflection served to enhance effective teaching, learning and empathetic skills. Understanding student perceptions during initial clinical experiences is crucial for improving educational programs (Hung et al. 2009), and participants reported that they felt more supported on clinical placement if they were welcomed and orientated sufficiently and noted that this occurred particularly if they spent more time with preceptors (Happell 2008). When placements were a positive experience, nursing students were able to shift their ideas and see consumers as people rather than just a diagnosis (Foster et al. 2021).

## 5 Discussion

This scoping review aimed to explore existing literature examining international nursing student experiences of mental health clinical placements within Australia. However, our search identified that there is no published literature regarding this phenomenon, highlighting a critical need for deeper understanding. Our findings reveal that mental health clinical placements play a transformative role in students’ development, particularly in advancing their skills, knowledge, and attitudes towards mental health care (Patterson et al. 2016; Perlman et al. 2017). Students generally report that placements help to bridge the gap between theoretical knowledge and practical application, enhancing their confidence and competence in working with individuals experiencing mental health issues (Foster et al. 2021; Zhang et al. 2021). However, most of the studies included in this review indicate that students experience negative emotions when commencing placements in a mental health nursing environment, such as anxiety and fear. Notably, these studies have been conducted in countries where students are fluent in local languages and customs; literature indicates that international students experience ‘culture shock,’ including language and communication challenges, stress in moving to an environment with major social and cultural differences, and experiencing racism (Graham 2024; Jeong et al. 2011; Mitchell et al. 2017). These experiences may be compounded in a mental health clinical environment, where nursing students already experience high rates of verbal abuse and racism (Dhari et al. 2025; Ferns and Meerabeau 2008).

Several additional issues emerged during mental health placements that caused concern for international nursing students, including the emotional demands of mental health work, the challenge of managing personal boundaries, and, in some cases, the stigma associated with mental health care. These concerns often lead to anxiety and apprehension (Acı et al. 2022; Alhamidi and Alyousef 2023; Demir and Ercan 2018; Karimollahi 2012; Song 2015) especially among those who may not have been adequately prepared for the interpersonal complexities and emotional intensity of the setting. International students may face heightened challenges due to language barriers, unfamiliarity with the local healthcare system, and differing cultural norms around mental health (Hansen et al. 2021). These factors can impede their ability to fully engage with patients and limit their learning opportunities, emphasising the need for tailored support mechanisms.

Despite positive experiences, students continue to express that mental health would not be an area of nursing that would be considered as their future career choice (Gonella et al. 2020; Lim et al. 2020; Shen et al. 2023; Zhang et al. 2021). Gonella et al. (2020) identified that there was no change in the desire to specialise in mental health after graduation even when students reported overall higher satisfaction of mental health placement in regards quality than other areas of nursing. Foster et al. (2021) echo these findings and point out that the development and greater understanding increased an interest in mental health nursing as a career. However, further research is required; for instance, a survey of 386 Norwegian nursing students (Kloster et al. 2007) found 6.2% (*n* = 24) expressed interest in working in mental health nursing at the beginning of their nursing program, whereas near the completion of their program this had risen to 12.7% (*n* = 49). In contrast, a survey of 1534 Italian nursing students (Matarese et al. 2019) found a persistently low rate of intent to work as mental health nurses both at the commencement and completion of their degree (3.3% and 5.3%, respectively).

The increasing reliance by both tertiary education providers and healthcare services nationwide on international nursing students underscores the importance of fostering supportive learning environments tailored to their unique needs and backgrounds. These students often bring diverse cultural perspectives on mental health and mental illness (Hansen et al. 2021), which may shape their initial attitudes and expectations of mental health clinical placements. This was evident in Mansouri and Darvishpour's (2024) study, where students held cultural beliefs that consumers were sinners, despite their clinical placement experience. Existing research has explored this phenomenon; for example, a qualitative study conducted by Subu et al. (2022) interviewed 15 nurses and 15 patients in Indonesia to explore religious and cultural perspectives on mental illness, finding themes of witchcraft, the supernatural, possession and the notion that mental illness was a “sinful or cursed” illness. Other global studies support these ideas of mental illness (for example, Endrawes et al. 2007; Morrison and Thornton 1999). Apart from these notions of the cause of mental illness, there are also global jurisdictions where mental illness is considered shameful, a punishment, and subject to high amounts of stigma that hinder help‐seeking (Dardas and Simmons 2015). Understanding these initial perspectives is essential, as they can influence students' adaptation to the mental health clinical setting and impact their overall learning experience, especially as they are entering into the healthcare study and learning environment in Australia.

This review also highlighted factors that could contribute to a more effective MH clinical placement experience. Key recommendations include providing pre‐placement orientation (Henderson et al. 2007), implementing mentorship programs with experienced mental health practitioners (Günaydin and Arguvanli Çoban 2021), and fostering a supportive and inclusive clinical environment that encourages open communication (Patterson et al. 2016; Perlman et al. 2017). For international students, these strategies could be further enhanced by cultural competency training and language support, helping them to feel more integrated within the clinical setting and better prepared to engage with diverse patient populations (Mitchell et al. 2017).

## 6 Limitations

The primary limitation of this scoping review was the inability to locate literature exploring the experiences of international nursing students in Australia attending mental health clinical placements. A comprehensive search strategy was designed to mitigate this risk; however, there appears to be a lack of literature exploring international student experiences of mental health clinical placements in Australia. Therefore, this review has attempted to apply existing literature exploring student experiences of mental health clinical placement to the international student perspective; however, this is also a strength as it indicates a substantial research gap that requires urgent attention.

## 7 Conclusion

There is a current and anticipated global shortage of mental health nurses. One strategy used to address the Australian nursing shortage has been to increase international student intake in pre‐registration nursing courses. However, this scoping review located no studies that have explored the experiences of international pre‐registration nursing students during clinical placement in mental health settings in Australia. It is well established that mental health nursing is not a popular career pathway for nursing students and that positive experiences during mental health placement can influence student decisions to pursue a career in this field. Therefore, we recommend research into the unique experiences of international students on mental health placement in Australia, with a focus on how to best support this student cohort. In the absence of any published research into international student experiences of mental health placement, we sought to map what is known about nursing students' experience of mental health placement throughout the world. While we have not been able to capture the unique experience of being a student in a mental health setting in a foreign country, our findings have highlighted various ways universities, clinical educators and clinicians can support students and make the experience of mental health placement more positive.

## 8 Relevance for Clinical Practice

This scoping review highlights the potential implications for both nursing education and mental health service capability. As international students become part of the nursing workforce, understanding and addressing the challenges experienced during mental health clinical placement is imperative for preparing confident, competent and engaged future clinicians. While mental health clinical placement continues to be pivotal in shaping students' skills, knowledge and attitudes towards consumers experiencing mental health conditions, for international students, the complexity of clinical placement is amplified by language, cultural nuances and adaptations. If left unaddressed, these challenges may carry into clinical practice, potentially affecting workforce integration and consumer care.

## Author Contributions

A.W., R.M. and A.S. devised the study methodology. A.W. conducted the search, and A.W., R.M. and A.S. performed screening, data extraction and charting collaboratively. A.W. wrote and revised the manuscript, and R.M. and A.S. revised the manuscript. All authors have reviewed the manuscript.

## Funding

The authors have nothing to report.

## Conflicts of Interest

The authors declare no conflicts of interest.

## Supporting information


**Data S1:** inm70221‐sup‐0001‐DataS1.docx.

## Data Availability

The authors have nothing to report.
