# Supplementary figures and images for: Pre‐Registration Nursing Student Experiences of International Mental Health Clinical Placement: A Scoping Review With Relevance to the Australian Context
Source: Int J Ment Health Nurs. 2026 Jan 27;35(1):e70221. doi: 10.1111/inm.70221 (PMC12835837; doi:10.1111/inm.70221)

**Appendix 1: Search Strategy.**

Medline:


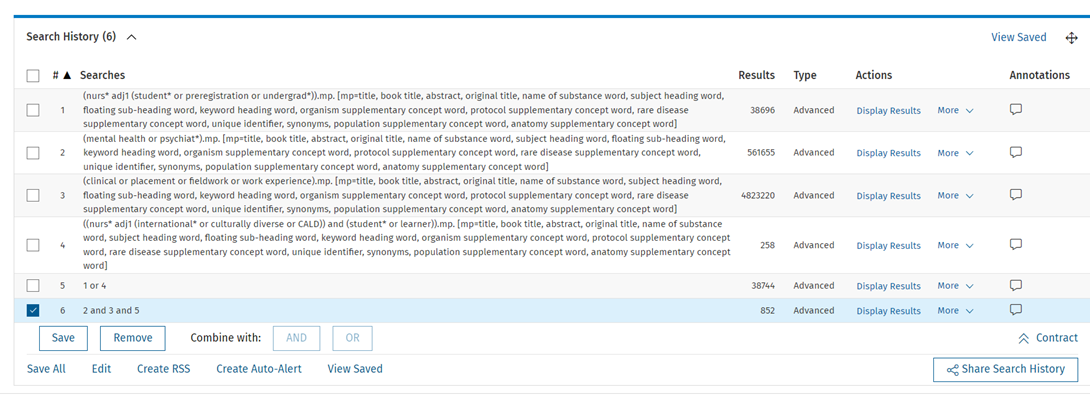


CINAHL:


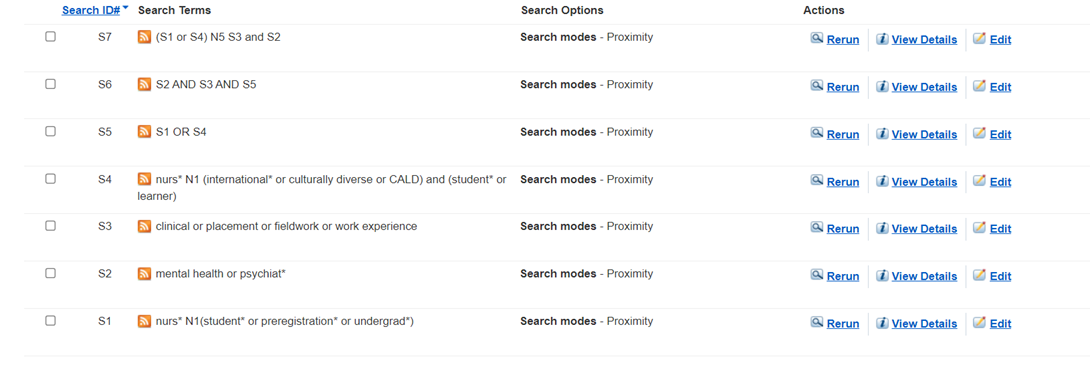


PsycINFO:


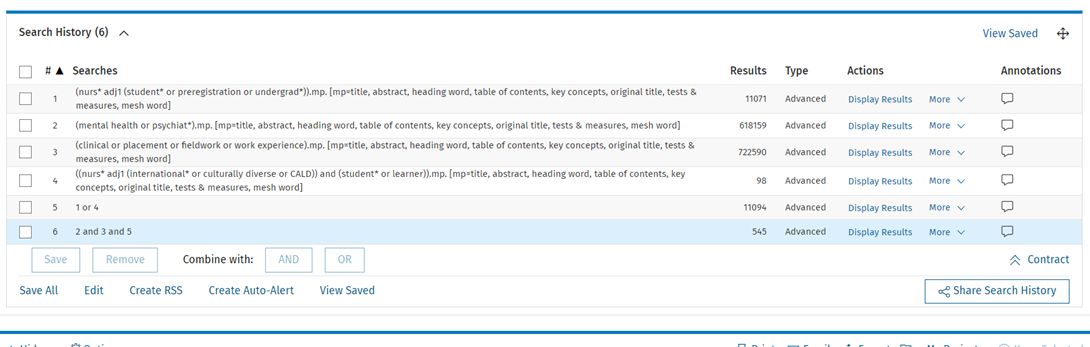

Supplement: Supplementary file 1 — Data S1: inm70221‐sup‐0001‐DataS1.docx. [file INM-35-0-s001.docx]
